# Classification of Critical Levels of CO Exposure of Firefigthers through Monitored Heart Rate

**DOI:** 10.3390/s21051561

**Published:** 2021-02-24

**Authors:** Raquel Sebastião, Sandra Sorte, José M. Fernandes, Ana I. Miranda

**Affiliations:** 1 Institute of Electronics and Informatics Engineering of Aveiro (IEETA) & Department of Electronics, Telecommunications and Informatics (DETI), University of Aveiro, 3810-193 Aveiro, Portugal; jfernan@ua.pt; 2 Centre for Environmental and Marine Studies (CESAM) & Department of Environment and Planning (DAO), University of Aveiro, 3810-193 Aveiro, Portugal; ssss@ua.pt (S.S.); miranda@ua.pt (A.I.M.)

**Keywords:** physiological data, heart rate, CO exposure, exposure classification, firefighters health

## Abstract

Smoke inhalation poses a serious health threat to firefighters (FFs), with potential effects including respiratory and cardiac disorders. In this work, environmental and physiological data were collected from FFs, during experimental fires performed in 2015 and 2019. Extending a previous work, which allowed us to conclude that changes in heart rate (HR) were associated with alterations in the inhalation of carbon monoxide (CO), we performed a HR analysis according to different levels of CO exposure during firefighting based on data collected from three FFs. Based on HR collected and on CO occupational exposure standards (OES), we propose a classifier to identify CO exposure levels through the HR measured values. An ensemble of 100 bagged classification trees was used and the classification of CO levels obtained an overall accuracy of 91.9%. The classification can be performed in real-time and can be embedded in a decision fire-fighting support system. This classification of FF’ exposure to critical CO levels, through minimally-invasive monitored HR, opens the possibility to identify hazardous situations, preventing and avoiding possible severe problems in FF’ health due to inhaled pollutants. The obtained results also show the importance of future studies on the relevance and influence of the exposure and inhalation of pollutants on the FF’ health, especially in what refers to hazardous levels of toxic air pollutants.

## 1. Introduction

Every year, firefighters (FFs) suppress thousands of wildfires that burn millions of hectares. In Europe, an average of 65,000 fires occurs annually, corresponding to 500,000 ha of wild land and forests being burnt, and more than 85% of the burnt area being located in the Mediterranean region [1]. A decrease in the occurrence of forest fires is not expected in the upcoming decades, and not only are fires occurring in new areas, previously almost untouched in Europe, but they are also happening at an unprecedented frequency, intensity and growth rate in areas where fires have occurred for millennia [2,3,4]. Forest fires seriously contribute to environmental pollution at local and regional scales and pose a threat to human health [5].

FFs are exposed to a high concentration of carbon monoxide (CO), particulate matter (PM) and volatile organic compounds (VOC), among other pollutants. Several adverse health effects have been reported in the literature, including lack of oxygenation to organs and tissues (measured by the increase in exhaled CO after smoke exposure), impaired respiratory function or increased risk of cancer [6,7,8,9,10].

According to an Australian study, exposure to CO concentrations of 200 ppm (parts per million) for 2–3 h will lead to light headaches in a healthy person; exposure to 400 ppm for 1–2 h may cause nausea, headache, and sickness; exposure to CO concentrations above 800 ppm may cause a confused state, asphyxia, convulsions or even coma [11].

Besides smoke inhalation, FFs face other hazards including extreme fire behaviour, heat, stress, fatigue, high temperatures, and reduced visibility [8]. Faint or disorientation episodes, due to smoke inhalation or impaired visibility conditions, are common in firefighting operations [8,12,13]. Moreover, FFs perform heavily physical work [14], and are exposed to long periods of stress during their fighting activities. Stress diagnosis is extremely difficult, namely because defining stress itself is still a matter of debate and the accepted process relies on a psychological evaluation. Notwithstanding the challenge of trying to identify stress in real environments where such information can be useful, it was demonstrated that there is an association between stress and coronary diseases [15,16,17].

The case of firefighting in forest fires, where FFs are exposed to stressful situations, is particularly relevant. A study in the United States in 2007, revealed that 45% of the deaths among FFs had a cardiovascular cause [18,19]. Results from [20] indicate that the most stressful FF tasks might be differentiated by the morphological measures over the electrocardiogram (ECG), namely the most stressful (e.g., car accidents, fires) and the least stressful (administrative services). Taking into consideration that CO is considered one of the air pollutants with a strong impact on the safety of the FF in the terrain [5,13], we worked on estimating CO inhalation with derived respiration through ECG and with the detection of changes in the heart rate (HR) of FFs related with alterations in CO exposure [21,22].

The main goal of this work is to go further, characterizing the HR of FFs based on occupational exposure standard (OES) values defined for the CO pollutant and classifying different levels of CO exposure, from data obtained during firefighting. Classifying CO exposure based solely on HR data has the advantage of reducing the complexity of models, maintaining the ability of real-time classification. Moreover, as it relies only on one predictor, it avoids the need for data synchronization. Ultimately, a decision-support system may be designed, classifying, in real-time, CO exposure based on HR data gathered with minimally invasive equipment. Such a system would help to create the safer and more successful management of FFs by preventing health potential effects, namely intoxication and/or damage to respiratory function.

The remainder of this paper is organized as follows: Section 2 describes the experimental setup, the equipment, the methodology of data collection and physiological monitoring, as well as the methods used for data analysis. In Section 3 the results are presented and discussed. Final remarks and future prospects in this field of research are introduced in Section 4.

## 2. Methodology

This section describes the experimental setup, the equipment, the procedures to gather physiological and environmental data, and presents the methods applied for characterizing HR data and for classifying CO exposure. The different methods used to analyse the data were implemented in MATLAB R2019b [23].

### 2.1. Experimental Setup, Data Collection and Physiological Monitoring

The research team adopts the principles of the Helsinki Declaration, revised in October 2013, defining the Ethical Principles for Medical Research in Humans. In this context, and under the approval of the Ethics Committee of the University of Porto (CEUP), Portugal, the research team involved in this study had always considered FFs’ health as the first concern. All the procedures were explained to the voluntary participants, as well as being informed that there were no risks involved in participating and that they could decline from participating in the study at any time.

The data used in this study were collected in 2015 and 2019, in the region of Gestosa, central Portugal, during experimental fires. The slope and vegetation of the different burnt fields presented the ideal conditions for this purpose [8,12,13,24]. A characterization of the experimental fields can be found in [21,22].

**Figure 1:**
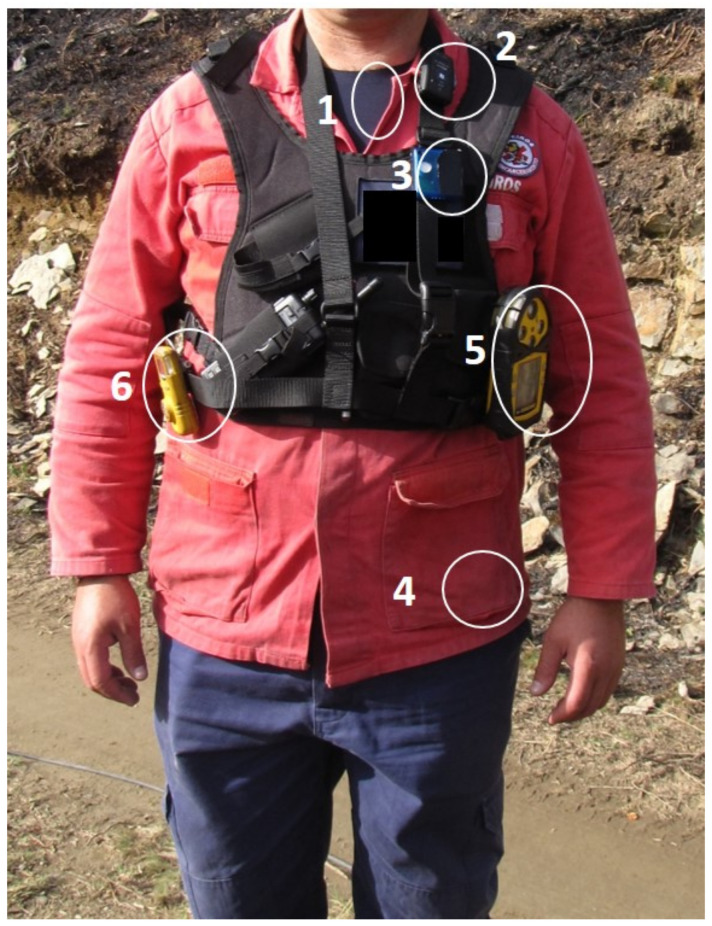
VR2 equipment: (1) VitalJacket^®^ t-shirt; (2) GPS; (3) FREMU; (4) Smartphone; (5) GasAlert Extreme CO; (6) GasAlertMicro 5 PID.

FFs were using the VR2 system [25]. This system consists of a GPS, a FREMU (First Responder External Measurement Unit), a smartphone and a VitalJacket^^®^^ t-shirt [26]. FREMU is a piece of equipment for environmental data monitoring (air temperature, atmospheric pressure, and CO concentration). The CO sensors were measured in-continuum at a 60 s intervals during the experiments. The sensor measures CO in air up to 500 ppm or 1000 ppm with a resolution of 0.1 ppm or 1 ppm, and was calibrated before the fire experiments using a 100 ppm CO calibration gas. The VitalJacket^®^ t-shirt is a wearable monitoring system able to gather body temperature, triaxial accelerometer and ECG data, with a frequency of 500 samples per second, without compromising the FF activities. All the monitoring equipment, for physiological and environmental data collection, were minimally invasive, as can be observed in Figure 1. The equipment for environmental monitoring was placed over the FF protective clothing, while the VitalJacket^®^ t-shirt was worn underneath to allow ECG collection (with wet gel Ambu BlueSensor electrodes, size L [27], since these have the advantage of allowing monitoring for long-term periods.) In addition, for comparison purposes (out of scope of this work), the CO concentration was also monitored using the GasAlert Extreme CO sensor from BW technologies.

Monitoring environmental and physiological data in similar firefighting conditions is difficult to accomplish. For more than a half dozen of years, we had only the possibility of monitoring data on 7 FFs during experimental field burns, 4 FFs in 2015 and 3 FFs in 2019. Seven male FFs, healthy and physically active, were selected as participants. To allow a combined analysis, the data collected were synchronized and were also verified if the variables of interest had any acquisition problem (such as sensor faults or errors). During this phase, and considering the purposes of the present study, only data from FFs that had been directly exposed to smoke were selected, resulting on a dataset from three FFs, with ages between 20 and 28 y.o. (FF3 from the experimental fires performed in 2015 and FF6 and FF7 from 2019). It must be pointed out that data synchronization was needed for the purpose of model classification construction. Afterwards, for classifying new data, synchronization is not needed since the classification model relies only on the HR data as the predictor. This allows us to provide real-time classification of CO exposure of FFs when in firefighting scenarios.

Despite the reduced final number of FFs under study, ECG and exposure to CO data were monitored for a period of 5 h (on average), resulting in a total of 92,945 samples.

### 2.2. CO Exposure Levels

To evaluate the CO exposure of FFs, we relied on the OES values, defined by the American Conference of Governmental Industrial Hygienists (ACGHI), respecting the: Threshold Limit Value–Time-Weighted Average (TLV–TWA) for 8 h, Threshold Limit Value–Short-Term Exposure Limit (TLV–STEL) for 15 min and peak limit.

According to these OES values, the CO exposure (COexp) levels were divided into 4 levels (“L1”, “L2”, “L3” and “L4”):

- L1: 0 ppm ≤ COexp < 25 ppm;
- L2: 25 ppm ≤ COexp < 200 ppm;
- L3: 200 ppm ≤ COexp < 400 ppm;
- L4: COexp ≥ 400 ppm.

During the experimental fires, none of the three FFs under this study was exposed to CO concentrations of level “L4” (above 400 ppm). Thus, in the further analysis, only 3 levels of CO exposure were considered.

### 2.3. ECG Data

The ECG data of each FF was recorded through the Vital Jacket^®^ t-shirt, with a sampling rate of 500 Hz, during the operational scenarios. The ECG signals are affected by noise, such as skin–electrode interference (low frequency noise, which is amplified by motion, movements and respiratory variation), power lines (with frequencies of 50 Hz) and electronic devices (high frequency noise) interferences. To attenuate the effects of noise and improve the quality of the signal, the raw ECG was low-pass filtered at a cut-off frequency of 40 Hz, as the useful band of frequencies for these research purposes, without clinical relevance, varies between 0.5 Hz and 40 Hz. The fundamental frequencies for the QRS complex, which is composed by Q, R and S waves, are below 30 Hz, and for the P-wave and T-wave components are below 20 and 10 Hz, respectively [28]. Afterwards, the baseline wander was removed with a moving average filter.

Using the filtered ECG signal, we computed the number of beats per minute, the HR, by first detecting the R peak locations and computing the distance between R peaks locations Dist(R,R), and then dividing the number of samples within one minute by the distance between R peaks, according to the following equation:

(1) $$HR=\frac{60\;SampleRate}{Dist(R,R)}$$

**Figure 2:**
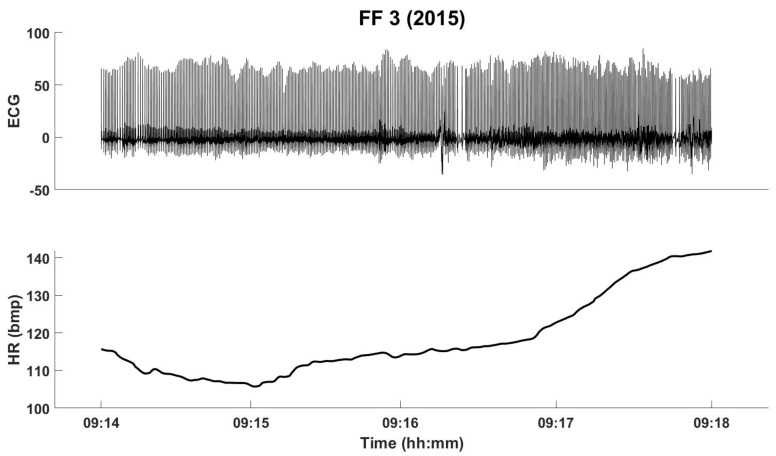
The filtered ECG and the HR for FF3, from the fire experiments in 2015 (for four minutes).

**Figure 3:**
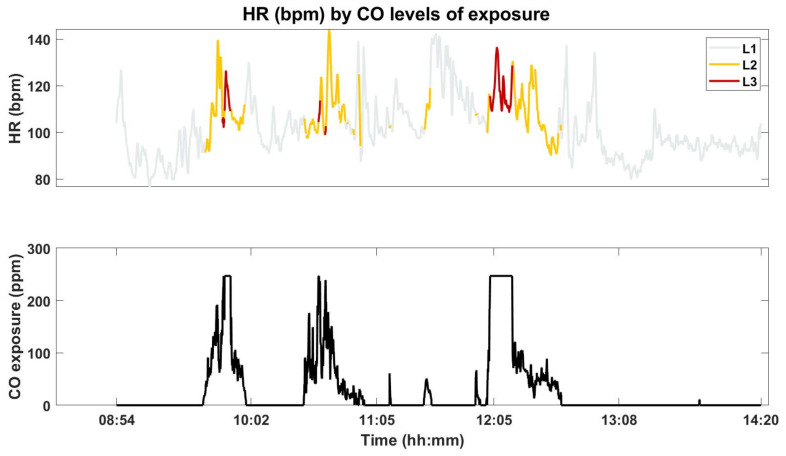
The FF7’s HR according to the CO levels of exposure (**top**) and the measured CO concentration (**bottom**).

Therefore, the HR is a time series representing the number of beats per minute, as shown in Figure 2 and Figure 3 (top). Figure 2 shows 4 min of the collected ECG and the respective HR, for FF3. Measured HR values of the three FFs were, most of the times, above 100 beats per minute. During intense activities, the HR should not exceed 70% of the maximum HR, which depends on the age. Assuming an averaged maximum HR of 220, we can state that FFs’ HR values were between the maximum limit for normal HR at rest (100) [29] and the maximum limit for intense activities (154).

### 2.4. Classification Method for CO Levels of Exposure

When relating FFs’ exposure to CO and the physiological condition using HR is of paramount importance to monitor synchronously and per each FF individual. Table 1 presents the number of HR data and the CO exposure duration according to the levels of CO exposure, for the three FFs. It can be observed that the number of HR samples associated with “L3” levels of CO exposure, as well as the exposure duration, are considerably smaller than for the remaining levels. Level “L3” is the minority class, while level “L1” is the majority class. Indeed, “L1” accounts for 82.3% of the data, “L2” for 14.1% of data and “L3” only for 3.6% of the collected data.

Figure 3 shows the HR of FF7, according to the CO levels of exposure (top) and the CO measured concentration (bottom).

From a visual analysis, not all the differences in the CO concentrations seem to be related to changes in the HR. However, in most cases, the FF changes in the CO concentration exposures are associated with higher values of HR. The CO concentration exposure appears to have more impact in the variance of the HR rather than on the amplitude itself, inducing changes, some abrupt, in HR data.

To explore if the HR data differ according to the different CO exposures, we performed a box plots analysis on the differences between the HR medians of the 3 CO levels, for all FFs in the study.

To test the differences between exposure level groups, we first applied the Lilliefors test to decide if data come from a normal distributed family. The HR data for the three FFs failed to be normal distributed.

Therefore, to analyse the differences between the 3 CO levels for the three FFs under study, we applied the Kruskal–Wallis (KW) Test, a nonparametric test, that allows to decide if the samples from the 3 levels of CO are originated from the same distribution, by comparing the mean ranks of the 3 CO levels groups. This test was performed with 3 different combinations of data, in order to ensure that samples from the 3 levels of CO exposure were independent. Therefore, for each of the KW tests, each group of CO is drawn from a different FF.

For each of those tests, in the case of differences between the 3 exposure level groups, we further analyse those through multiple comparisons between the groups. For that, we use the multicompare function from MATLAB, which, besides returning the pairwise comparison results based on the statistics outputted from the KW test, also allows for an interactive graphical multiple comparison of the groups, displaying the mean rank estimates for each group and comparison intervals.

Afterwards, for classification purposes, to surpass the drawback of class imbalance of the data, we produced Gaussian noisy replicates of the “L2” and “L3” classes, in order to get the same number of samples for each CO level of exposure (data as also scaled to be between the interval [0, 1] in order to allow inter-subject analysis). With this over-sampling method, we had a total of 230,001 samples, with 76,667 samples from each CO exposure level.

To decide on the best method for classifying CO levels through the HR collected, we created an ensemble of learners for classification with data from the three FFs, using bagging, adaptive boosting and random undersampling boosting algorithms. Then, using the best method to fit the ensemble with the HR data to CO levels, we estimated the misclassification rate and computed the confusion matrix using 10-fold cross-validation. Finally, this ensemble was trained with 70% of the HR data, and the remaining 30% of the data, held out for testing, was used on the model to make predictions.

### 2.5. Evaluation Metrics

The accuracy of a model translates its performance by the relation between the number of corrected classifications by the total number of data examples. To further evaluate the performance of the classifier, the confusion matrix was calculated, allowing to compute quality metrics as *Precision* and *Recall*. In a classification problem with more than 2 classes, for a given class, we can describe the true positives (TP) as the number of correct predictions, the false negatives (FN) as number of examples of that class that were predicted as belonging to other classes, and the false positives (FP) as the number of false alarms, i.e., the number of examples of the other classes that were predicted within that class. Therefore, the *Precision* is defined as the ratio between the correct predictions (TP) and all the predictions of a given class (TP+FP), while *Recall* is the ratio of correct predictions (TP) and all the examples that actually belong to that class (TP+FN). In the case of both metrics get high values, then the different classes are properly handled by the classifier. Combining both, the $F_{1}$ measure is defined as the harmonic mean of the afore mentioned metrics.
$Precision=\frac{TP}{TP+FP}$ , $Recall=\frac{TP}{TP+FN}$ and $F_{1}=2\frac{Precision\;Recall}{Precision+Recall}$

## 3. Results and Discussion

**Figure 4:**
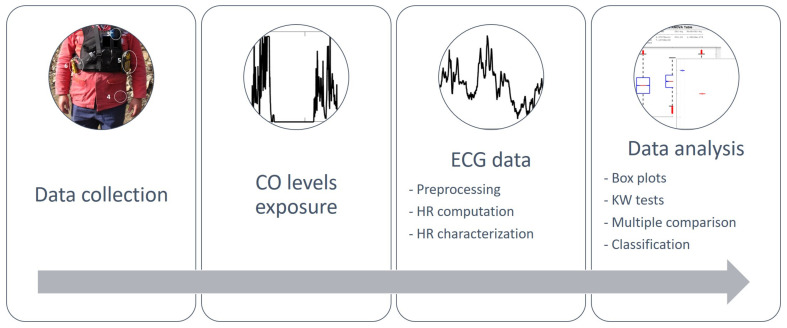
Data analysis workflow.

As described before, the workflow process for data analysis consisted of several steps (as Figure 4 illustrates), from the collection of data until the evaluation of the results, including the definition of CO levels of exposure, the preprocessing of ECG data, the computation of HR, and the box plots of HR, the KW test, the multiple comparison tests to evaluate the differences in FFs’ HR between the 3 levels of CO exposure and the classification of CO levels through the HR of the three FFs under study.

**Figure 5:**
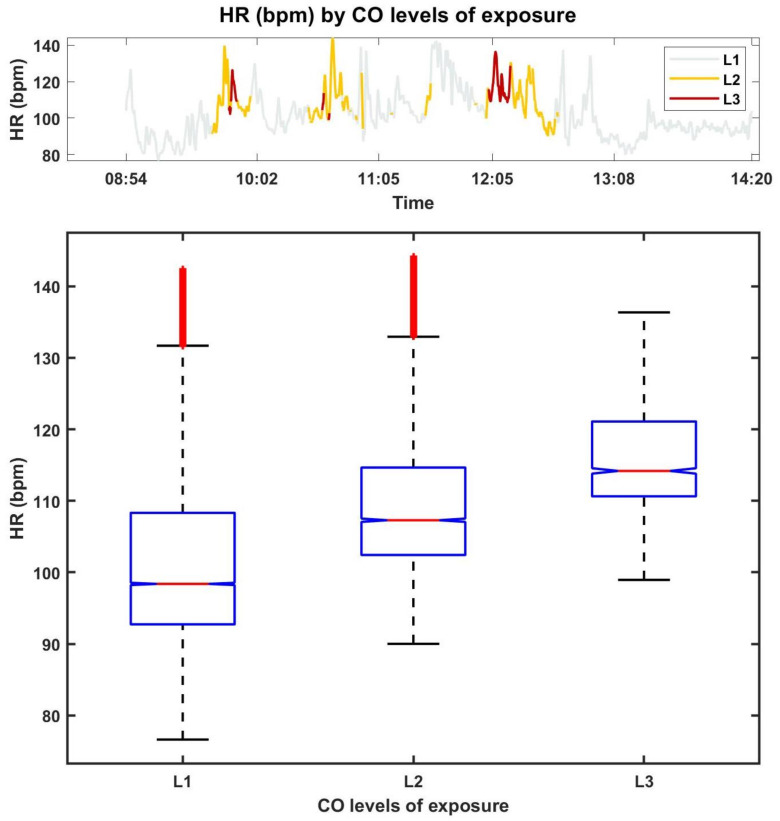
Box plots of the HR of FF7, per exposure level.

The box plots, obtained with data from FF7 (as in Figure 3) are shown in Figure 5.

The top figure provides the HR, according to CO levels of exposure (similar to the top of Figure 3) and the bottom figure presents the box plots. Through the analysis of the box plots we can observe that the HR associated with CO levels “L2” and “L3” present higher median values, which are due to the sudden changes in the CO concentrations. This is clearly illustrated for FF7 (see also Figure 3) by sharp burst in CO concentration coupled to “L1” and higher levels. It can also be observed that the higher the CO level of exposure, the less the variance in the HR, which can be justified by the smaller number of samples for this class when compared with the other classes. For the remaining FFs the same behaviour was verified.

**Figure 6:**
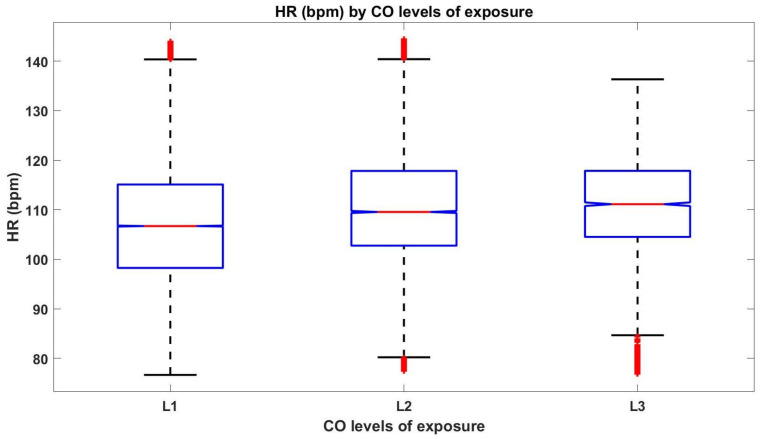
The box plots for HR data of the three FFs considered in this study, per CO exposure level.

The box plots in Figure 6 visually presents the summary statistics of the HR of the three FFs for the levels of CO exposure, showing that the greater the CO level, the higher the median number of beats per minute (the same conclusion is also verified when the HR of the FFs is analysed individually, as shown in Figure 5). It can also be observed, that, despite being the level with lesser data, the HR within the higher level of CO (“L3”) presents several outliers.

Figure 6 also points out, with 95% confidence, that the true HR medians of the 3 CO levels are different. To further confirm this, we applied the KW test on 3 different combinations of data, to ensure samples independence, as follows:

- Combination 1: HR data during “L1” level of exposure from FF1, HR data during “L2” level of exposure from FF2 and HR data during “L3” level of exposure from FF3;
- Combination 2: HR data during “L1” level of exposure from FF2, HR data during “L2” level of exposure from FF3 and HR data during “L3” level of exposure from FF1;
- Combination 3: HR data during “L1” level of exposure from FF3, HR data during “L2” level of exposure from FF1 and HR data during “L3” level of exposure from FF2.

For each of the 3 KW tests, the returned *p*-values (<0.05) indicate that, at a significance level of 5%, the null hypothesis that the HR from the three levels of CO exposure that come from the same distribution is rejected. As the KW tests allowed us to conclude that the median values of HR from the 3 levels of CO exposure are significantly different, we performed multiple comparisons tests to reveal which from the 3 groups are significant different from the others, for the three combinations of data.

**Figure 7:**
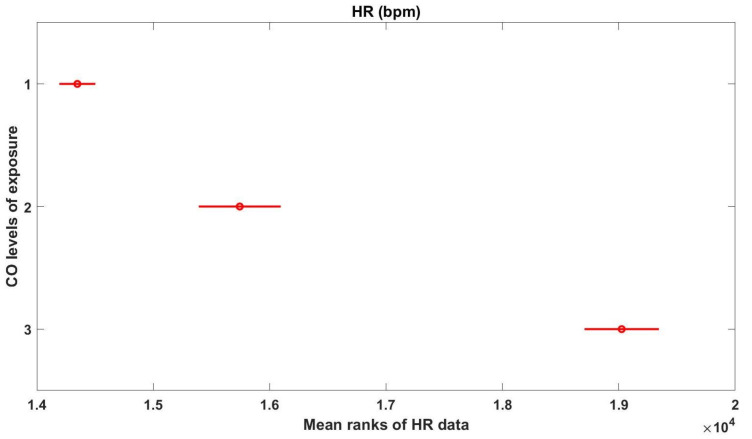
Multicomparison graphics for the mean ranks of HR, for each level of CO exposure.

Figure 7 presents the estimates of the mean rank orders of HR values, and 95% confidence comparison intervals, for the different CO exposure groups in combination 1. As the comparison intervals, for the three groups, do not overlap with each other’s, one can conclude that the three groups of CO exposure have mean ranks significantly different from each other’s. The same conclusion can be drawn from data within combination 2. Moreover, and analysing the returned matrix of pairwised comparison results, one can confirm these results (*p*-values < 0.05), indicating that median HR values for levels “L1” and “L2”, for levels “L1” and “L3” and for levels “L2” and “L3”, are significantly different, at a significance level of 5%, for both combinations 1 and 2.

With respect to data in combination 3, the groups from “L2” and “L3” of CO exposure are not significantly different from each other (*p*-value = 0.8425 > 0.05), but both are significantly different from “L1” group (*p*-values < 0.05)).

Therefore, considering these results, the classification of the different levels of CO measured during the firefighting experiments was performed on data after over-sampling. At first, to decide on the best method for classifying CO exposure levels, it was constructed a predictive classification ensemble using all the replicated predictor variables in HR data (230,001 samples). After optimization, the results suggested that the best method was bagging, with random predictor selections at each split (random forest).

Therefore, the misclassification rate and confusion matrix were estimated, using 10-fold cross-validation, obtaining an estimate cross-validated classification error of 6.9%. The obtained confusion matrix, presented in Table 2 shows, for all the 3 CO levels, high values of true positives (correct predictions), displayed in the principal diagonal of the matrix, and small values (when compared to those) of FP and FN. For each level, the number of true negatives (TN), is also high: 152,179, 141,683 and 150,276, for “L1”, “L2” and “L3” levels, respectively. It can also be observed that label “L1” presented a higher number of FN. As shown in Figure 6, breathing in high levels of CO can induce increased HR; however, physical exercise during firefighting also raises the HR, and the higher number of FN in the lower CO level may be due to exercise.

Finally, an ensemble of 100 bagged classification trees was trained using 70% of the replicated data (161,001 samples). The remaining 30% of data was used to test the ensemble (69,000 samples). Both test and train sets were constructed preserving the original class distribution.

For each CO level, the precision, recall and $F_{1}$ are presented in Table 3. The average obtained values are above 90%, which are acceptable results, showing the ability to classify CO levels of exposure. It is worthwhile to notice that results for the “L3” are slightly higher when compared to the remaining CO levels. This is due to the fact that “L1” is the minority class, and therefore, had a higher number of replicates.

The obtained accuracy of 91.9% is reinforced by the high values for the precision, recall and $F_{1}$ metrics, which indicate a good performance of the classifier, validating its capability to classify CO levels of exposure scores using HR data.

## 4. Conclusions and Further Research

Changes in HR can be induced by various motives, such as exercise or physical activities, a decrease in oxygen in breath, emotional stimulus or anxiety and stress states. In a previous work, we found out that changes in HR were associated with the inhalation of CO. Based on this, we proposed to characterize HR data according to different levels of CO exposure. The obtained results sustain that distinct levels of CO exposure differently affect HR, allowing the classification of CO levels of FFs’ exposure in relation to monitored HR data.

The classifier, based on an ensemble of 100 bagged classification trees, presented an overall accuracy of 91.9% and an average precision, recall and $F_{1}$, for “L1”, “L2” and “L3” levels, of 92.7%, 91.9% and 91.7%, respectively. These results show that is acceptable to classify the CO levels of exposure of FFs through HR monitored with minimally-invasive equipment.

Despite the limitations of the available data, the obtained results demonstrate the feasibility of a classification system to allow for the identification of hazard circumstances. The major advantage of such a system relies on the possibility to online access and analyse ECG data and derived CO exposure levels, through a communication protocol, providing real-time support on the health of FFs and allowing a better management of the teams involved in a firefighting scenario. Since CO exposure is classified, based solely on HR data, there is no need for data synchronization, which is a great advantage for real-time uses.

These results allow the identification of physiological correlates of CO exposure and can be further integrated into a decision support system.

This work shows the importance of future studies on the relevance and influence of the exposure and inhalation of pollutants on the FF’s health. The collection of more data, as well as other methods to embrace imbalanced data, are future steps that need to be accomplished.

## Figures and Tables

**Table 1:** Number of HR samples and exposure duration, per FF, as a function of CO exposure (COexp) levels.

COexp Levels	Time (min) (# HR Samples)			
	FF3 (2015)	FF6 (2019)	FF7 (2019)	Total
L1: 0 ≤ ppm < 25	246.8 (26,411)	249.4 (25,912)	240.0 (24,344)	736.2 (76,667)
L2: 25 ≤ ppm < 200	15.4 (1646)	42.8 (4454)	67.9 (6887)	126.1 (12,987)
L3: 200 ≤ ppm < 400	1.3 (138)	12.1 (1258)	18.7 (1895)	32.1 (3291)
L4: ppm ≥ 400	0	0	0	0
Total	263.5 (28,195)	304.3 (31,624)	326.6 (33,126)	894.4 (92,945)

**Table 2:** Confusion matrix of CO exposure level classification using HR from the three FFs under study.

		Predicted CO Exposure Level		
		L1: 0 ≤ ppm < 25	L2: 25 ≤ ppm < 200	L3: 200 ≤ ppm < 400
	L1: 0 ≤ ppm < 25	62,301	11,475	2891
COexp level	L2: 25 ≤ ppm < 200	909	75,591	167
	L3: 200 ≤ ppm < 400	246	176	76,245

**Table 3:** Precision, recall and $F_{1}$ measure for classifying CO levels through HR.

CO Level	Precision	Recall	$F_{1}$ Score
L1: 0 ≤ ppm < 25	98.0	77.7	86.7
L2: 25 ≤ ppm < 200	84.6	98.5	91.0
L3: 200 ≤ ppm < 400	95.4	99.4	97.4
Average	92.7	91.9	91.7

## Data Availability

Data is not available due to privacy and ethical restrictions.
